# Impact of Counterions on the Electronic Structure and Optical Properties of Water-Soluble Au_25_ Clusters

**DOI:** 10.1021/acs.jpca.6c00570

**Published:** 2026-05-08

**Authors:** Mohit Verma, Sami Malola, Hannu Häkkinen

**Affiliations:** † Department of Physics, Nanoscience Center, 4168 University of Jyväskylä, Jyväskylä FI-40014, Finland; ‡ Department of Chemistry, Nanoscience Center, University of Jyväskylä, Jyväskylä FI-40014, Finland

## Abstract

Ligand-stabilized gold nanoclusters have raised significant interest due to great advances in their atom-precise design for a range of functionalities for catalysis, optoelectronic applications, bioimaging, and nanomedicine. Theory and computational investigations can greatly help to understand the effects from clusters’ environment to their electronic structure and optical excitations. Here, we have performed a systematic density functional theory study on the impact of large counterions on the electronic structure of small water-soluble gold (Au_25_) clusters. We show that counterions not only stabilize cluster isomers as previously shown [Chem 2021, 7, 2227–2244] but also modulate excitation energies and redistribute oscillator and rotatory strengths among dipole-allowed transitions, significantly altering the photophysical behavior of the nanoclusters. These findings help to understand the photophysics of clusters in realistic solution-phase conditions advancing further design efforts for applications.

## Graphical abstract

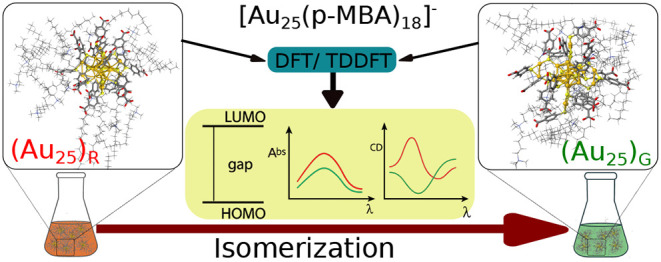

## Introduction

Metal nanoclusters with atomically precise compositions occupy a unique position between molecular systems and plasmonic nanoparticles. These clusters, typically from a few to a couple of hundred metal atoms surrounded by a ligand shell, exhibit discrete, molecule-like electronic levels, sharp optical transitions, and size-dependent redox behavior. These atomically precise, ambient-stable nanostructures have been termed as ″superatoms″, which is a useful theoretical concept helping to understand their electronic structure, optical response, and chemical reactivity.^1–3^ Because of their controllable structure–property relationships, such clusters have been exploited in applications spanning sensing, imaging, and catalysis where tunability of excited states and redox levels is critical.^4–8^

Among atomically precise gold nanoclusters, the thiolate-protected [Au_25_(SR)_18_]^−^ (SR = thiolate) remains the most thoroughly characterized and widely studied cluster. Its single-crystal structure reveals a centered icosahedral Au_13_ core encapsulated by six Au_2_(SR)_3_ polymeric units that form a well-defined metal/ligand interface.^9,10^ The crystal structure directly confirmed the ″core–shell″ structural motif from early theoretical predictions that stated that gold atoms in Au_*x*_(SR)_*y*_ clusters can be in two chemical oxidation states (neutral, metallic at the metal core, and oxidized between the thiolates in the gold–thiolate shell).^11^ Even before the groundbreaking experiments on the precise structure of this compound, high-resolution mass spectrometry revealed its chemical stability^12^ and density functional theory (DFT) calculations predicted its electronic stability as an eight-electron closed-shell superatom in 1S^2^1 P^6^ configuration by delocalized Au­(6s) electrons in the metal core.^1,13^ This early research was then followed by numerous experimental and theoretical characterizations of the clusters’ spectroscopic and chemical properties.^10,14–18^ The charge state of the cluster directly governs its optical and electronic response: oxidation or reduction induces measurable shifts in absorption and alters emission lifetimes, as shown by ultrafast spectroscopy.^14^ Time-dependent DFT calculations reproduced the characteristic absorption bands and clarified their origin in frontier orbital transitions, as well as the influence of ligand field and dielectric environment on transition energies.^3,16,17^ In addition to dielectric effects, interactions with the surrounding environment, such as solvent or ligand binding, can also influence the structural stability of gold nanostructures. Previous studies have shown that these interactions can lead to structural rearrangements and stabilization of different configurations.^19,20^ The combination of known atomic structure, tunable charge states, and stable fluorescence underpins its widespread use as a model cluster for understanding excited-state processes and structure–property correlations in atomically precise nanoclusters.

Although the crystal structure of [Au_25_(SR)_18_]^−^ with phenylethylthiolate (PET) ligands^9,10^ has served as the standard structural model for interpreting most experimental observations, several studies have shown that the protecting gold–thiolate shell can adopt alternative arrangements. An early indication came from the work of Lopez-Acevedo and Häkkinen^21^ who used methylthiolate (MET, SCH_3_) as a simplified ligand to examine possible rearrangements around the Au_13_ core. They proposed that the frequently detected [Au_21_(SR)_14_]^−^ fragment in ESI-MS experiments may originate from a structure in which the icosahedral core undergoes a subtle reorganization, allowing the terminating sulfur atoms of selected RS–Au–SR–Au–SR units to bind directly to neighboring core atoms. Liu and coworkers later reported a related theoretical structural motif using the same MET ligand model.^22^ Their calculations reinforced the idea that the gold–thiolate units can reorganize to form alternative metal–ligand connectivities that remain energetically competitive with the conventional arrangement. Together, these studies suggested that the ligand shell is capable of flexing and reorganizing under certain conditions, giving rise to low-energy isomeric forms even when the icosahedral Au_13_ kernel remains largely intact.

During the past few years, evidence on a new low-energy topological isomer of [Au_25_(SR)_18_]^−^ was first presented from a predictive DFT study by Matus Cortes et al.^23^ in 2020. It was followed by the experimental discovery in 2021 of such an isomer in gas-phase mass spectrometry and ion mobility study^24^ which showed two isomeric structures of [Au_25_(SR)_18_]^−^ with clearly distinct ion mobility cross-sections and the isomer with the large cross-section matching the predicted geometry.^24^ Xie’s group in 2021 demonstrated that reversible interconversion between two such isomers indeed exists in aqueous solution, by using *para*-mercaptobenzoic acid (*p*–MBA) as the ligand. They showed that the conversion between the cluster isomer ″red″, (Au_25_)_*R*_ (due to the color of its solution phase) and ″green″ (Au_25_)_*G*_ can be toggled by coupling/decoupling CH···π interactions between cetyltrimethylammonium (CTA^+^) counterions and the *p*–MBA phenyl rings.^25^ The measured optical absorption of the green isomer matched the computed absorption with the predicted structure.^23^ The optimized structures of these two isomers^25^ are shown in (Figure 1), highlighting the distinct ligand-shell arrangements that underpin their different optical behaviors. The CTA^+^ ions adsorb onto, and rigidify, the nanocluster’s outer environment, forming a double-layer shell that electronically couples to the ligand/metal interface. This counterion sheath thus emerges as an active element that can steer isomer populations under realistic solution conditions. Despite these insights, the extent to which counterion binding restructures the nanocluster electrostatics and how that, in turn, governs frontier orbital separations and optical selection rules remained unresolved.

**Figure 1:**
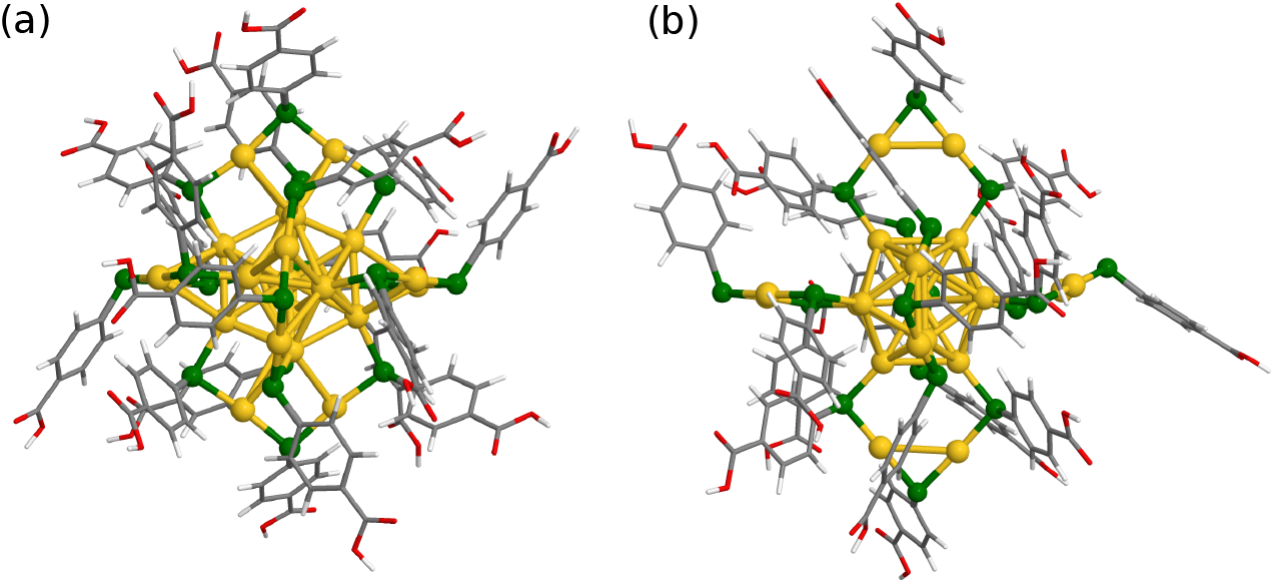
Optimized structures of the two [Au_25_(*p*-MBA)_18_]^−^ isomers: (a) the (Au_25_)*_R_* isomer and (b) the (Au_25_)*_G_* isomer. Gold atoms are shown in yellow, sulfur in green, carbon in gray, oxygen in red, and hydrogen in white. The structures are plotted from the DFT-optimized structures reported in ref 25.

In this study, we investigate how such interactions between the counterions and the clusters influence the electronic and optical properties of the (Au_25_)_*R*_ and (Au_25_)_*G*_ isomers in solution. Our results based on density functional theory (DFT) reveal that CTA^+^ not only drives isomer formation but also modulates excitation energies and redistributes oscillator and rotatory strengths among dipole-allowed transitions, significantly altering the photophysical behavior of the nanocluster. These findings highlight the critical role of counterions in both structural reorganization and optical tuning of metal nanoclusters, offering deeper insight into their behavior under realistic solution-phase conditions, which is important for the design of new responsive, chiroptically active nanocluster-based materials and devices.

## Computational Methods

All calculations were carried out within the framework of DFT using the GPAW package,^26^ which employs the projector augmented wave (PAW) method on a real-space grid. To account for solvation effects, an implicit solvent environment was included and the solvent parameters (for water) were set to the values used in a previous work^27^ where the model is described in detail. Exchange–correlation interactions were described within the generalized gradient approximation (GGA) using the Perdew–Burke–Ernzerhof (PBE) functional.^28^ All real-space calculations were done with a grid spacing of 0.20 Å. Scalar relativistic effects, which are essential for gold clusters, are inherently included in the PAW data sets used in GPAW. Spin polarization was not considered, and all calculations were performed in a spin-unpolarized framework. The initial structures of both (Au_25_)_*R*_ and (Au_25_)_*G*_ containing 24 CTA^+^ counterions were taken from molecular dynamics simulations in our previous work.^25^ To obtain a total cluster charge of −1, we removed the six farthest CTA^+^ ions from each structure, leaving 18 CTA^+^ ions around the Au_25_(p-MBA)_18_ cluster. The resulting charge states used in the calculations were −1 for the protonated p-MBA system, −19 for the fully deprotonated system, and −1 for the deprotonated p-MBA system with 18 CTA^+^ counterions. The optimized structures are shown in Figure 2a,b. The structures of all systems were fully optimized with a grid spacing of 0.2 Å until the maximum residual force on every atom was below 0.05 eV/Å, ensuring well-converged ground-state structures. Electronic occupations were described using a Fermi–Dirac distribution with a smearing width of 0.05 eV. The optical absorption and circular dichroism (CD) spectra were simulated through linear-response time-dependent density functional theory (LR-TDDFT) as implemented in GPAW^29^ under the implicit solvent environment. We used the PBE functional also in the kernel of the LR-TDDFT calculations. The calculated optical absorption and CD spectra were generated by applying a Gaussian broadening with a width of 0.1 eV to the individual electronic transitions. To interpret the nature of the excitations, we also performed dipole transition contribution map (DTCM) analysis.^30^ In DTCM analysis, the occupied and unoccupied states involved in a transition are projected onto the different structural regions of the cluster–Au­(core), Au–S interface, and the p-MBA ligands. This atom-resolved decomposition reveals how charge redistribution occurs during the excitation and enables us to identify whether a given feature arises primarily from metal–metal, metal–ligand, ligand–metal, or ligand–ligand interactions.

**Figure 2:**
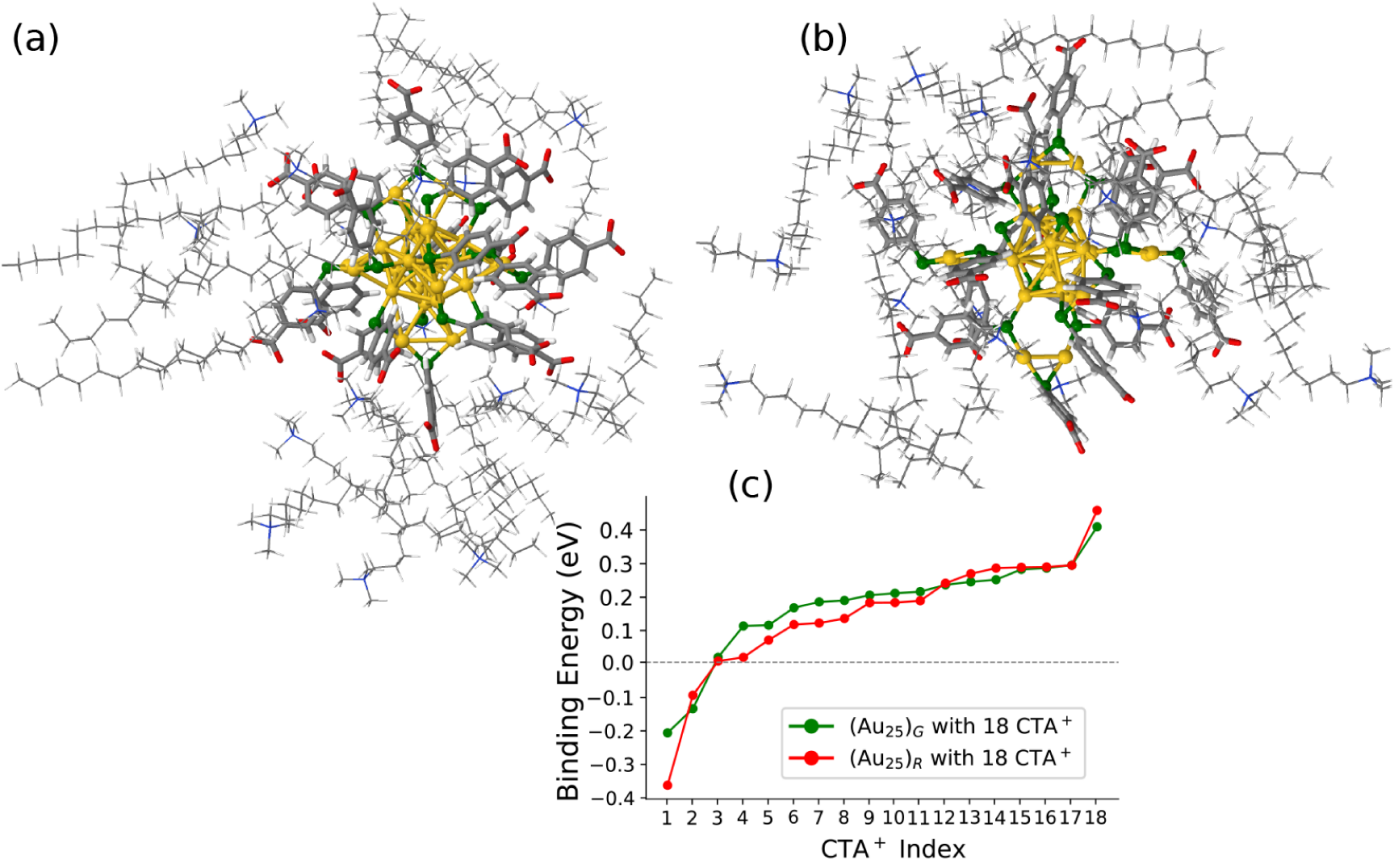
Optimized structures of the two [Au_25_(*p*-MBA)_18_]^−^ isomers with 18 CTA^+^ counterions: (a) the (Au_25_)*_R_* isomer and (b) the (Au_25_)*_G_* isomer. Gold atoms are shown in yellow, sulfur in green, nitrogen in blue, carbon in gray, oxygen in red, and hydrogen in white. (c) Binding energy profile of the 18 CTA^+^s for both isomers (positive energy means CTA^+^s are binding, and negative energy means not binding).

To investigate the interactions between the retained CTA^+^ ions and the cluster, we computed the binding energy of each CTA^+^ using

$$E_{\mathrm{binding}}=E_{\left({\mathrm{CTA}}^{+}\right)}+E_{({\mathrm{Au}}_{25}\left(p-\mathrm{MBA}{)}_{18}+17{\mathrm{CTA}}^{+}\right)}-E_{({\mathrm{Au}}_{25}\left(p-\mathrm{MBA}{)}_{18}+18{\mathrm{CTA}}^{+}\right)}$$

The results show that for both the isomers (Au_25_)_*R*_ and (Au_25_)_*G*_, among the 18 CTA^+^ ions, 15 exhibit positive binding energies (i.e., favorably bound), two have negative binding energies, and one has a binding energy close to 0 eV (Figure 2c).

## Results and Discussion

### Electronic Structure

We first analyze the atom-projected densities of states (PDOS) for the two isomers, (Au_25_)_*R*_ and (Au_25_)_*G*_, under three charge compensation conditions: protonated p-MBA, deprotonated p-MBA, and in the presence of 18 CTA^+^ (Figure 3). Across both isomers, deprotonation produces a clear redistribution of the ligand-derived PDOS. In the protonated clusters, the ligand states appear mainly at lower energies, well separated from the frontier region, where the Au–S interface and Au core states dominate. Upon deprotonation, the ligand manifold broadens and shifts to higher energies, partially overlapping with Au–S-derived states and thus creates four distinct ligand bands as shown in Figure 3b,e. This upward shift is consistent with the added negative charge on the −COO^–^ groups, which increases ligand-interface coupling and raises the energy of several ligand-localized levels. When 18 CTA^+^ ions are introduced, the ligand bands shift to slightly higher energy, additional ligand-associated features appear at even lower energies, and the overall ligand/Au–S hybridization becomes stronger.

**Figure 3:**
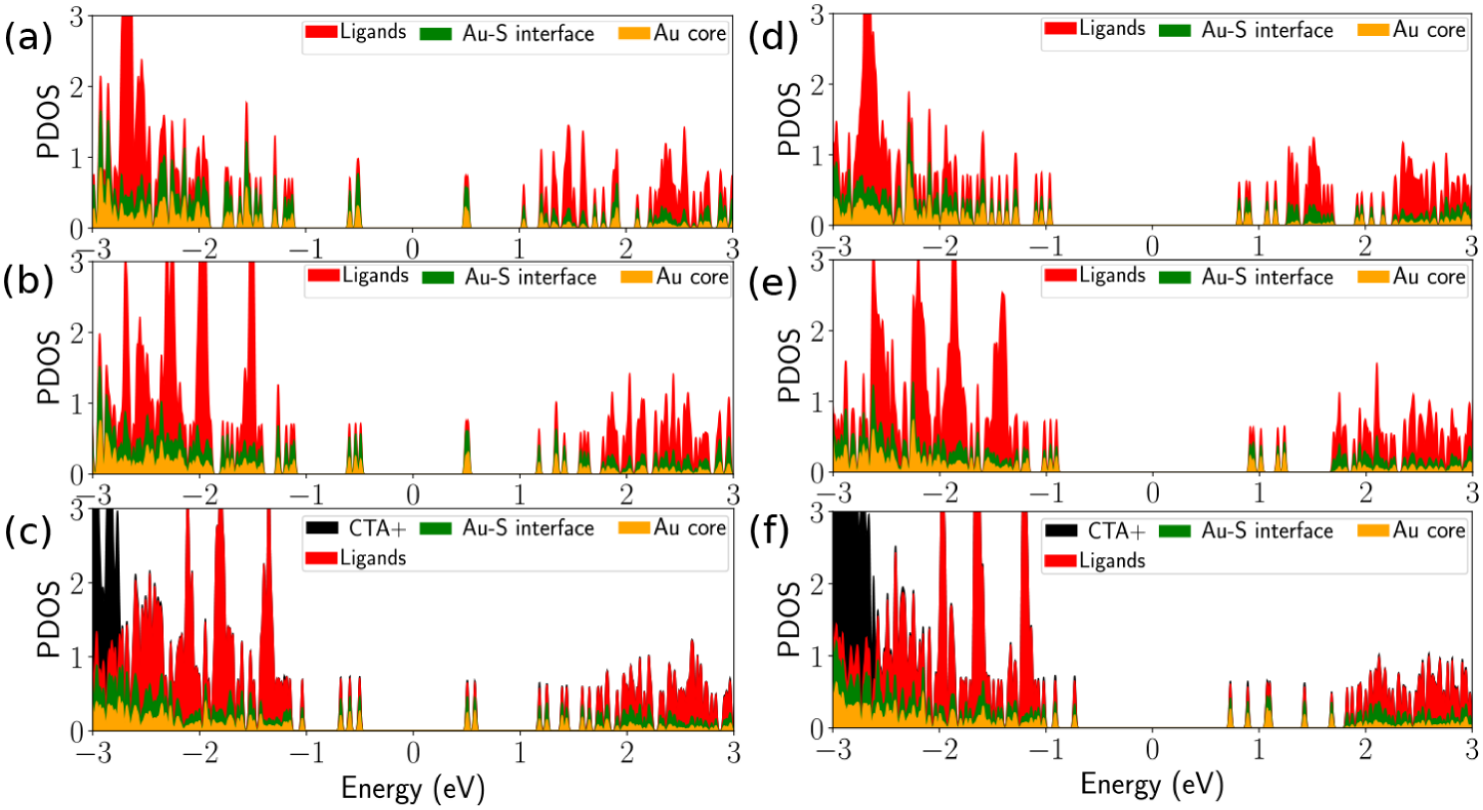
Projected densities of states (PDOS) for (Au_25_)*_R_* (a-c) and (Au_25_)*_G_* (d-f) under ligands protonated (a,d), ligands deprotonated (b,e), and with 18 CTA^+^ ions (c,f).

The two isomers respond differently near the frontier levels. In (Au_25_)_*R*_ (Figure 3a–c), states around the HOMO and LUMO originate primarily from the Au–S interface and extend up to the frontier region. Consequently, the HOMO–LUMO gap remains small (≈1.0 eV) and is almost unchanged across protonated, deprotonated, and 18 CTA^+^-compensated conditions (Table 1). The presence of CTA^+^ does not alter the gap significantly but does refine the electronic structure: the triply degenerate P-like HOMO set and the D-like LUMO levels become more clearly separated, and the LUMO shifts slightly upward, causing a minor increase in the gap (≈10 meV).

**Table 1:** Kohn–Sham (KS) Radial Potential Statistics for (Au_25_)_(*R*/*G*)_ with *P*-MBA, in Three Charge-Compensation Conditions^a^

Isomer	Condition	Mean *V*_KS_ (2.6 Å)	Mean *V*_KS_ (*r*_outer_)	Δ*V* (eV)	Gap (eV)
*R* (*r*_outer_ = 5.1 Å)	Protonated	–22.23	–16.90	5.33	0.996
	Deprotonated	–21.67	–16.31	5.36	0.986
	+18 CTA^+^	–17.90	–13.96	3.94	1.007
*G* (*r*_outer_ = 5.8 Å)	Protonated	–22.91	–15.54	7.37	1.778
	Deprotonated	–22.38	–15.08	7.30	1.819
	+18 CTA^+^	–21.99	–15.65	6.34	1.461

a Mean of potential (*V*_KS_, in eV) is reported at *R* = 2.6 Å (inner Au core layer) and at the outer Au–S interface region (*r*_outer_). Δ*V* is the potential energy difference. Gap is the HOMO–LUMO energy gap.

The (Au_25_)_*G*_ isomer (Figure 3d–f) behaves quite differently. With protonated or deprotonated ligands, the HOMO–LUMO gap is larger (1.78–1.82 eV), reflecting a depletion of states around the Fermi level. When 18 CTA^+^ are added, the gap decreases to 1.46 eV. This difference arises because in (Au_25_)_*G*_, the remaining part of the HOMO outside the Au core has stronger ligand character than in (Au_25_)_*R*_, while the LUMO remains more associated with the Au–S shell. Electrostatics changes at the surface and therefore shifts the HOMO and LUMO unequally, reducing their separation.

The radial Kohn–Sham potential, *V*_KS_(*r*) (Figure 4), helps explaining these PDOS observations. Both the isomers with protonated *p*-MBA show a deep well in the Au core region (*r* = 2.6 Å), followed by a second minimum in the Au–S layer (*r*  ≈ 5–6 Å), and then a steady rise toward the vacuum. Deprotonation does not affect the features of the curve but it slightly raises the overall potential as seen in Figure 4a,b. For both isomers, CTA^+^ shows different variations in the curve. For the (Au_25_)_*R*_ isomer, the presence of CTA^+^ ions smooths the potential profile, effectively removing the second minimum in the Au–S layer. In contrast, for the (Au_25_)_*G*_ isomer with CTA^+^, the potential curve not only retains the overall trend but also shows a deeper (more negative) second minimum in the Au–S region (see Table 1). This indicates that the (Au_25_)_*G*_ isomer experiences a stronger stabilization at the interface. The more negative potential also suggests enhanced electron density localization around the Au–S layer in the (Au_25_)_*G*_ isomer compared to the (Au_25_)_*R*_ isomer when CTA^+^ ions are included. We define the potential difference (Δ*V*) between the inner Au core and the Au–S surface as

$$\Delta V=\left|\left\langle V_{\mathrm{KS}}\left(2.6Å\right)\right\rangle -\left\langle V_{\mathrm{KS}}\left(5-6Å\right)\right\rangle \right|$$

**Figure 4:**
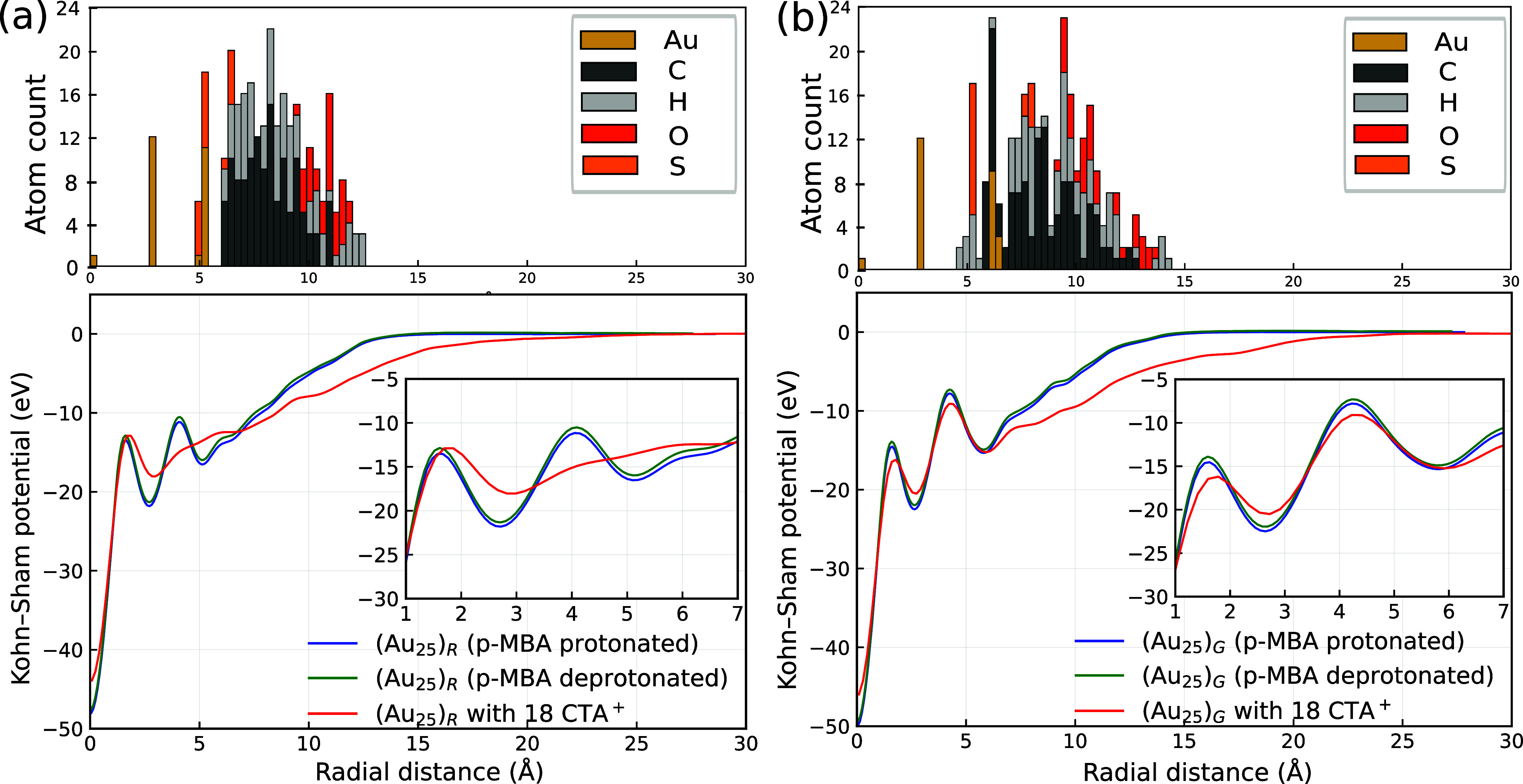
Calculated Kohn–Sham radial potentials, *V*_KS_(*r*), for (a) (Au_25_)*_R_* and (b) (Au_25_)*_G_* isomers under protonated ligands, deprotonated ligands, and with 18 CTA^+^. The upper panels show the corresponding radial distribution of atoms.

For (Au_25_)_*R*_, Δ*V* is about 5.3–5.4 eV with protonated or deprotonated ligands and shifts to 3.9 eV when CTA^+^ counterions are present (Table 1). Despite this reduction in confinement, the HOMO–LUMO gap remains nearly constant at ≈1.0 eV. This robustness arises because the three P-type HOMO states lie cleanly above the ligand band and move rigidly with the rest of the superatomic levels, leaving the gap unaffected by changes in the surface potential.

In contrast, (Au_25_)_*G*_ shows a deeper potential contrast: Δ*V* is 7.3–7.4 eV with protonated or deprotonated ligands, decreasing to 6.3 eV with CTA^+^ counterions. This decreased Δ*V* corresponds directly to weaker radial confinement and is mirrored by the HOMO–LUMO gap narrowing from ≈1.8 eV to ≈1.5 eV. Protonation alone alters Δ*V* only slightly (7.37 → 7.30 eV), producing a negligible change in the gap (≈0.04 eV). The strong correlation between Δ*V* and the gap in (Au_25_)_*G*_ reflects the fact that one of its P-type HOMO states mixes with the ligand band and is therefore sensitive to electrostatic changes at the shell.

Of the 18 CTA^+^ molecules, 11 form the first adsorbed layer on the cluster.^25^ We performed another set of calculations in which we built both isomer structures by removing the 7 farthest CTA^+^. To examine the role of the alkyl tail, we also replaced each remaining CTA^+^ with its headgroup, tetramethylammonium (TMA^+^; ${\mathrm{NC}}_{4}H_{12}^{+}$ ). We computed the PDOS and Kohn–Sham (KS) potential profiles for both models: 11 TMA^+^ and 11 CTA^+^ molecules (see Figure S2). For the (Au25)_*R*_ isomer, the HOMO–LUMO gap stays near 1.0 eV in all cases (0.986 eV deprotonated, 1.016 eV with 11 TMA^+^, 1.007 eV with 11 CTA^+^), showing little sensitivity to surface screening. For the (Au25)_*G*_ isomer, the effect is stronger: the gap drops from 1.819 eV (deprotonated) to 1.531 eV (11 TMA^+^) and to 1.398 eV (11 CTA^+^).

The distinct behavior of the P-type HOMO states highlights the structural origins of this difference. In (Au_25_)_*R*_, all three P levels lie cleanly above the ligand band, keeping the HOMO decoupled from the ligand states. In (Au_25_)_*G*_, however, one P level drops into the ligand band and mixes with it, giving the HOMO a strong ligand contribution. As a result, shifts in the ligand bands induced by counterions directly affect the HOMO in (Au_25_)_*G*_, contracting the gap, whereas the gap in (Au_25_)_*R*_ remains essentially unchanged.

### Optical Properties

The absorption spectra of (Au_25_)_*R*_ and (Au_25_)_*G*_ (Figure 5) were calculated using LR-TDDFT with protonated and deprotonated ligands as well as with 18 CTA^+^ counterions. We note that a weak low-energy peak is obtained for (Au_25_)_*R*_, arising predominantly from the HOMO–LUMO transition; however, this contribution was not considered further in the present analysis. Therefore, for (Au_25_)_*R*_, three major absorption peaks were identified at approximately 588 nm (P_1_), 440 nm (P_2_), and 403 nm (P_3_) in the protonated state. Upon deprotonation, the first band shifts to 561 nm, and the higher-energy features become less intense. In the cluster with 18 CTA^+^ counterions, the lowest peak appears near 593 nm, and the second band around 491 nm slightly strengthens, reflecting the reorganization of the unoccupied shell states.

**Figure 5:**
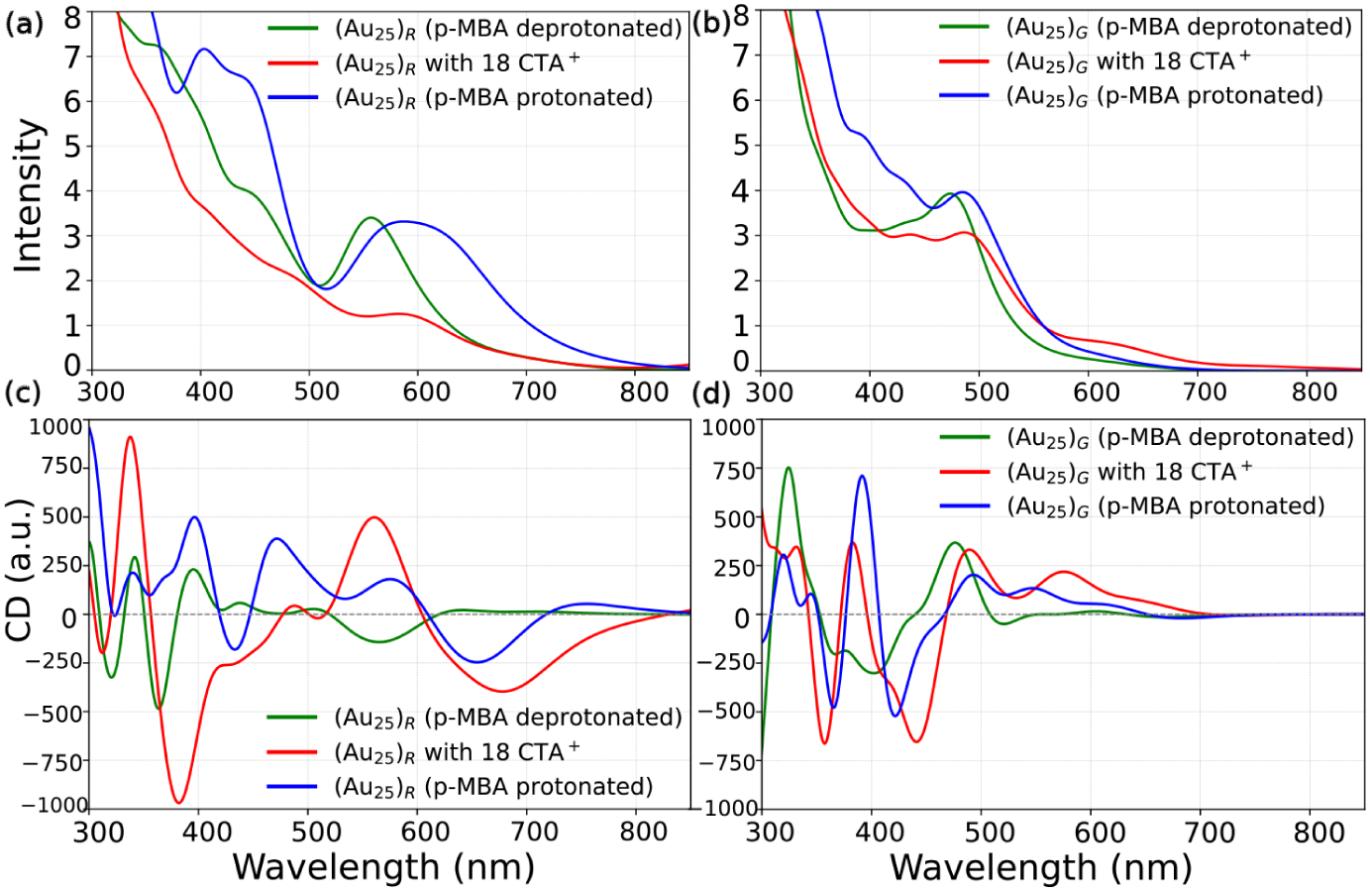
Calculated optical absorption spectra and circular dichroism (CD) spectra for (a,c) (Au_25_)*_R_* and (b,d) (Au_25_)*_G_* isomers under protonated, deprotonated, and with 18 CTA^+^.

For (Au_25_)_*G*_, the corresponding peaks appear at 486 nm (P_1_), 432 nm (P_2_), and 343 nm (P_3_) under protonated conditions. Deprotonation causes a blue shift to all three peaks (to 475, 362, and 312 nm, respectively), consistent with a reduction in the number of orbitals contributing to the excitation. When 18 CTA^+^ are binding, the main peak returns to 492 nm, accompanied by a pronounced shoulder near 438 nm, confirming partial recovery of orbital coupling between the core and ligand framework. For a direct comparison with the experiment, the calculated spectra of both (Au_25_)_*R*_ and (Au_25_)_*G*_ were red-shifted by 0.4 eV (as was done in ref 25), consistent with the known underestimation of excitation energies at the PBE level. For (Au_25_)_*R*_ (Figure S1a), the protonated system shows higher intensity in the 450–600 nm region than observed experimentally, while the deprotonated system introduces a clearer minimum around 550–600 nm, bringing the spectral shape closer to the experimental curve. The spectrum with 18 CTA^+^ further improves the agreement at longer wavelengths (>600 nm), where it follows the gradual decay of the experimental profile more closely. For the (Au_25_)_*G*_ (Figure S1b), a similar trend is observed. The protonated spectrum overestimates the low-energy intensity, while the deprotonated system reduces the intensity and shifts spectral weight toward shorter wavelengths, improving the agreement in the 450–650 nm region. The inclusion of 18 CTA^+^ produces a smoother decay and a slightly red-shifted profile relative to the deprotonated case, which better follows the experimental curve at longer wavelengths. Although no single configuration reproduces the experimental spectrum exactly, the main trends are captured, showing that the calculated changes originate from physically meaningful variations in the electronic structure due to ligand charge state and counterion screening.

To gain deeper insight into these spectral features, we use the dipole transition contribution map (DTCM) analysis (Figures 6, 7), which separates each optical excitation into its constituent electron–hole contributions and thus helps us assign the main bands to metal–metal, metal–ligand, or ligand-based excitations. In the DTCM plots, red and blue contours indicate strengthening and screening contributions, respectively. For (Au_25_)_*R*_, P_1_ is built from a limited manifold of transitions that includes near-frontier metal-centered states together with noticeable participation from ligand-derived states. Across protonated, deprotonated, and 18 CTA^+^ structures, the DTCM indicates that largely the same metal/ligand manifold contributes to this excitation, with the main change being a redistribution of relative weights rather than the appearance of new dominant transitions. At higher energies, the absorption features of (Au_25_)_*R*_ become increasingly collective. P_2_ is formed by contributions from several occupied states near the highest occupied levels and multiple low-lying unoccupied states, while P_3_ involves an even broader set of contributing excitations. Deprotonation and 18 CTA^+^ mainly broaden the contributing transition manifold and redistribute intensities, without changing the qualitative character of these higher-energy excitations. In contrast, (Au_25_)_*G*_ exhibits collective electronic contributions already for P_1_, with several comparable contributions from multiple occupied and unoccupied states near the frontier region. The higher-energy peaks of (Au_25_)_*G*_ show a strong dependence on protonation: upon deprotonation, P_2_ and P_3_ shift to higher excitation energies and the DTCM shows increased participation from higher-energy unoccupied states, whereas the presence of 18 CTA^+^ counterions partially reverses these changes, bringing both the peak energies and the dominant contributions closer to the protonated case.

**Figure 6:**
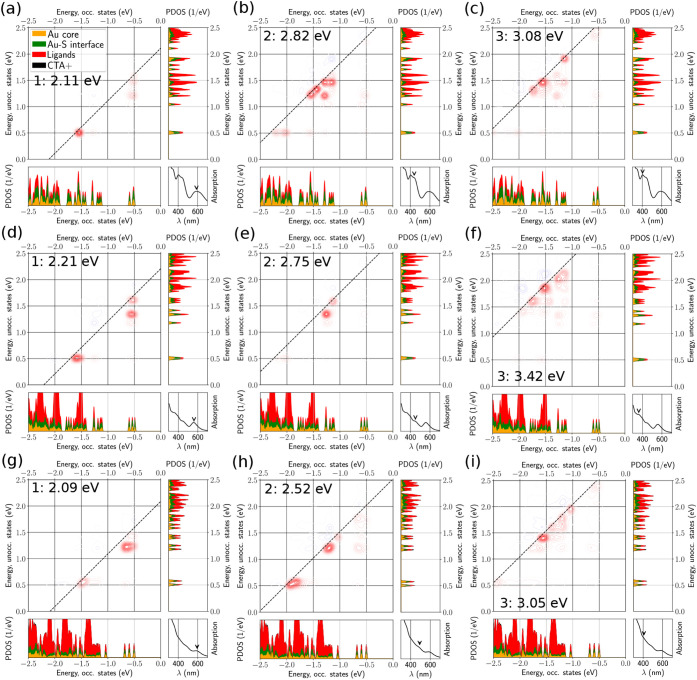
Dipole transition contribution map (DTCM) analysis of three absorption peaks of (Au_25_)*_R_* under protonated ligands (a,b,c), deprotonated ligands (d,e,f), and with 18 CTA^+^ (g,h,i).

**Figure 7:**
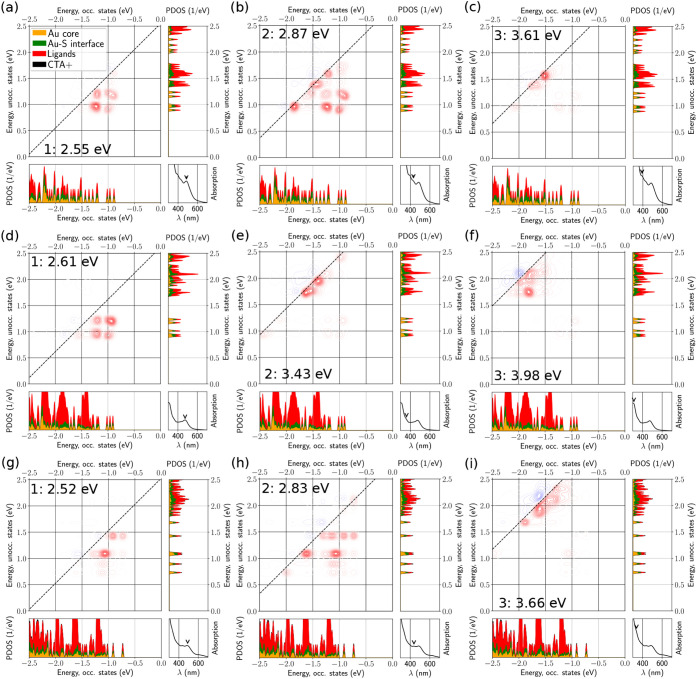
Dipole transition contribution map (DTCM) analysis of three absorption peaks of (Au_25_)*_G_* under protonated ligands (a,b,c), deprotonated ligands (d,e,f), and with 18 CTA^+^ (g,h,i).

The circular dichroism (CD) spectra of the (Au_25_)_*R*_ and (Au_25_)_*G*_ (Figure 5c,d) exhibit structured line shapes with multiple sign changes across the visible region, rather than the reported simple two-lobed (bisignate) pattern in clusters where chirality is introduced via chiral ligands or chiral ligand arrangements.^31–34^ Since p-MBA is an achiral ligand, the observed chiroptical response arises from symmetry breaking in the overall Au–S–ligand framework, where specific ligand orientations and interfacial geometries lead to a chiral environment that enables CD-active optical transitions. The CD spectra reported here are consistent with this picture, indicating that the magnitude and shape of the CD response are governed by ligand-shell asymmetry and its coupling to the electronic structure, rather than by the intrinsic chirality of the ligand molecules.

For (Au_25_)_*R*_, the CD signal is strongest in the 340–500 nm range, overlapping the P_2_ and P_3_ absorption regions, and its magnitude increases markedly upon inclusion of 18 CTA^+^. The enhanced CD intensities reach the same order of magnitude as those reported for the chiral Au_38_(PET)_24_ system^35^ with typical extrema in the order of 10^3^ in comparable CD units. This enhancement reflects a strengthening of the existing asymmetry in the ligand-shell environment and its coupling to the optically active transitions, rather than the introduction of new chiral elements. In contrast, (Au_25_)_*G*_ exhibits a weaker overall CD response: the signal decreases upon deprotonation and is only partially recovered in the presence of 18 CTA^+^. This muted sensitivity is consistent with the more stable and deeper Au–S interfacial potential in (Au_25_)_*G*_, which restricts ligand-shell reorganization and limits changes in the chiroptical response. Consequently, CTA^+^ binding stabilizes the electronic and structural configuration of (Au_25_)_*G*_ more efficiently, whereas (Au_25_)_*R*_ undergoes more pronounced optical and chiroptical reorganization under the same conditions.

The absorption spectra of the two Au_25_ isomers with 11 TMA^+^ and 11 CTA^+^ counterions (Figure S3a) show clear differences in how each isomer responds to changes in the CTA^+^ environment. For the (Au_25_)_*R*_, the spectral profiles retain the same key features across the different counterion models. All curves display a broad rise in intensity between 300 and 550 nm followed by a gradual decay toward 800 nm, and the positions of the weak shoulder near ∼450–470 nm and the broader feature around ∼580–600 nm remain nearly unchanged. The main effect of increasing CTA^+^ ions is a uniform increase in intensity at low energies, while the peak positions themselves remain stable. In contrast, the (Au_25_)_*G*_ (Figure S3b) shows more pronounced spectral evolution. The low-energy shoulder near ∼430 nm becomes smoother and slightly red-shifted with increasing CTA^+^ content, and the broad band around 480–500 nm progressively loses intensity. The overall profile flattens as the number of CTA^+^ ions increases, suggesting stronger perturbation of the frontier orbitals and enhanced screening effects in (Au_25_)_*G*_.

The CD spectra (Figure S3c,d) mirror these behaviors with additional chiroptical detail. For the (Au_25_)_*R*_, the negative band near ∼380 nm—followed by positive features around ∼550–600 nm—appears in all models with only modest changes in amplitude. The positions of these peaks remain essentially unchanged, again reflecting the robustness of the (Au_25_)_*R*_’s electronic response. In contrast, the (Au_25_)_*G*_ exhibits small but noticeable shifts in its main CD bands: the negative feature around ∼350 nm becomes slightly less intense with additional CTA^+^, while the positive band near ∼480–500 nm broadens and shifts marginally.

In summary, both isomers display a comparable first absorption feature (P_1_), built from near-frontier metal-centered states with noticeable ligand-derived contributions, while the higher-energy bands (P_2_–P_3_) respond very differently to protonation, deprotonation, and CTA^+^ binding. For (Au_25_)_*R*_, these chemical modifications lead to a redistribution and broadening of electronic transitions, accompanied by enhanced optical intensity and a strong increase in the CD response. In contrast, (Au_25_)_*G*_ shows more limited spectral changes, including modest blue shifts of P_2_ and P_3_ and weaker variations in CD intensity. These differences also connect to the origin of chirality in the two structures: the geometry of (Au_25_)_*G*_ already supports a well-defined chiroptical response that is only weakly modified by environmental changes, whereas (Au_25_)_*R*_ develops a stronger CD response primarily when the ligand environment is perturbed by protonation changes or CTA^+^ binding. This indicates that ligand arrangement and ligand–environment interactions play a more central role in generating chirality in (Au_25_)_*R*_, while (Au_25_)_*G*_ remains comparatively stable.

## Conclusions

This work establishes that ion pairing at the ligand–environment interface decisively governs both the structural and optical characteristics of Au_25_(SR)_18_. CH···π interactions between cetyltrimethylammonium (CTA^+^) and the aromatic p-MBA ligands not only drive the reversible interconversion between the (Au_25_)_*R*_ and (Au_25_)_*G*_ isomers but also reshape their underlying electronic landscape. Ground-state and time-dependent DFT analyses reveal that CTA^+^ adsorption modulates the Kohn–Sham potential across the Au–S interface, compresses the core–surface potential difference (Δ*V*), and redistributes oscillator strength among low-energy transitions. Although both clusters show similar shifts in the ligand PDOS upon deprotonation and CTA^+^ adsorption, their frontier orbitals respond in fundamentally different ways. In (Au_25_)_*R*_, the three P-type HOMO states remain cleanly separated from the ligand band, which preserves the HOMO–LUMO gap at ∼1.0 eV across all environments. In contrast, the (Au_25_)_*G*_ isomer contains a P-type HOMO state that merges with the ligand manifold, making its frontier levels more sensitive to changes at the shell. As a result, CTA^+^ binding lowers the interfacial Kohn–Sham potential and reduces the HOMO–LUMO gap by nearly 0.4 eV. These differences highlight that the electronic structure of (Au_25_)_*R*_ is more internally stabilized, whereas (Au_25_)_*G*_ is more strongly influenced by external electrostatics.

The distinct optical and chiroptical responses of the two isomers arise from how asymmetry is accommodated at the ligand–metal interface. In (Au_25_)_*R*_, modifications of the ligand charge state and counterion environment more readily distort the interfacial geometry, leading to pronounced changes in absorption line shapes and a strong enhancement of the CD response. This behavior indicates that optical activity in (Au_25_)_*R*_ is closely tied to environment-induced symmetry breaking within the ligand shell. In contrast, (Au_25_)_*G*_ maintains a more rigid interfacial arrangement, so variations in protonation and CTA^+^ binding produce comparatively smaller changes in both absorption and CD spectra. As a result, the chiroptical response of (Au_25_)_*G*_ is less sensitive to external perturbations, reflecting a more constrained coupling between structural asymmetry and optical activity.

Overall, the two clusters exhibit complementary forms of sensitivity. The (Au_25_)_*R*_ isomer maintains a stable HOMO–LUMO gap but displays a more flexible optical response, with its chiroptical features adjusting readily to changes in the ligand shell. The (Au_25_)_*G*_ isomer, on the other hand, shows a rigid optical signature but an electronically tunable frontier region. Ion pairing at the ligand-solvent boundary thus emerges as a powerful handle to reversibly control both the electronic structure and optical response of metal nanoclusters in solution. This interplay between counterion organization and frontier state alignment provides a mechanistic basis for electrostatically driven optical tuning, offering opportunities to engineer responsive, chiroptically active nanoclusters for future applications in sensing, photoactive interfaces, and molecular-scale optoelectronic devices.

## Supplementary Material



## Notes

Experimental vs calculated absorption spectra of (Au_25_)_*R*_ and (Au_25_)_*G*_; partial densities of states (PDOS) and Kohn–Sham radial potential profiles for (Au_25_)_*R*_ and (Au_25_)_*G*_ isomers under different counterion environments (TMA^+^ and CTA^+^); calculated optical absorption and circular dichroism (CD) spectra under varying counterion compositions; tabulated Kohn–Sham radial potential statistics, potential energy differences, and HOMO–LUMO gaps (PDF)
