# Supplementary material for: Impact of Counterions on the Electronic Structure and Optical Properties of Water-Soluble Au25 Clusters
Source: J Phys Chem A. 2026 May 8;130(20):3879–88. doi: 10.1021/acs.jpca.6c00570 (PMC13200178; doi:10.1021/acs.jpca.6c00570)
Supplement: Supplementary file 1 [file jp6c00570_si_001.pdf]

Supporting Information: Impact of Counterions on the Electronic Structure and Optical Properties of Water-Soluble Au_{25} Clusters

Mohit Verma,† Sami Malola,† and Hannu Häkkinen*,†,‡

†*Department of Physics, Nanoscience Center, University of Jyväskylä, FI-40014 Jyväskylä, Finland*

‡*Department of Chemistry, Nanoscience Center, University of Jyväskylä, FI-40014 Jyväskylä, Finland*

E-mail: hannu.j.hakkinen@jyu.fi

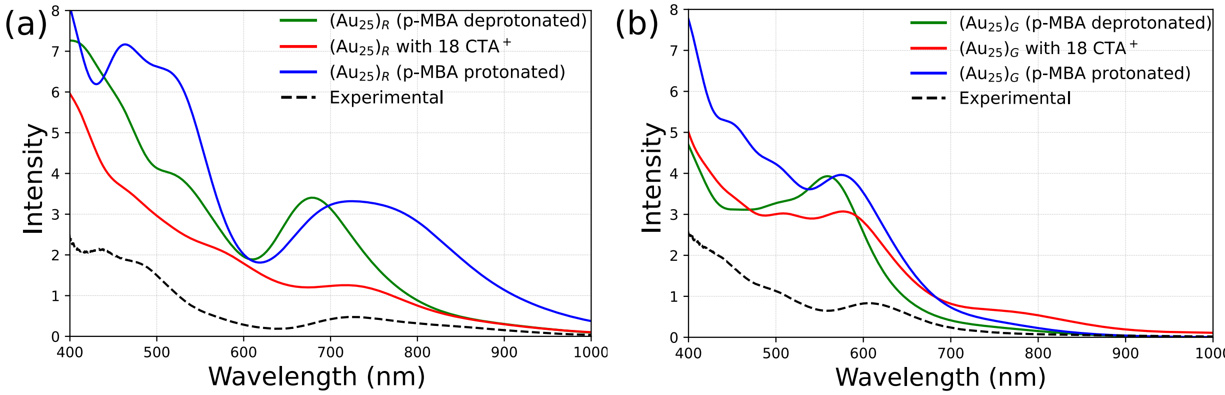

Figure S1: Experimental vs calculated optical absorption spectra for (a) $(Au_{25})_R$ and (b) $(Au_{25})_G$ isomers under protonated, deprotonated, and with 18 CTA^+. Calculated spectra are rigidly shifted by 0.4 eV towards lower energies. The experimental data is from ref. 25 in the main text.

Table S1: Kohn–Sham (KS) radial potential statistics for $(Au_{25})_{(R/G)}$ with *p*-MBA, in three counterion conditions. Mean of potential (V_{KS}) is reported at r = 2.6 Å (inner Au core layer) and at the outer Au-S interface region (r_{outer}). ΔV is the potential energy difference. Gap is the HOMO-LUMO energy gap.

Isomer	Condition	Mean V_{KS} (2.6 Å)	Mean V_{KS} (r_{outer})	ΔV (eV)	Gap (eV)
R (r_{outer} = 5.1 Å)	+11 TMA^+	−17.74	−13.62	4.12	1.016
	+11 CTA^+	−18.61	−13.70	4.91	1.007
	+18 CTA^+	−17.90	−13.96	3.94	1.007
G (r_{outer} = 5.8 Å)	+11 TMA^+	−22.03	−14.75	7.28	1.531
	+11 CTA^+	−22.08	−15.23	6.85	1.398
	+18 CTA^+	−21.99	−15.65	6.34	1.461

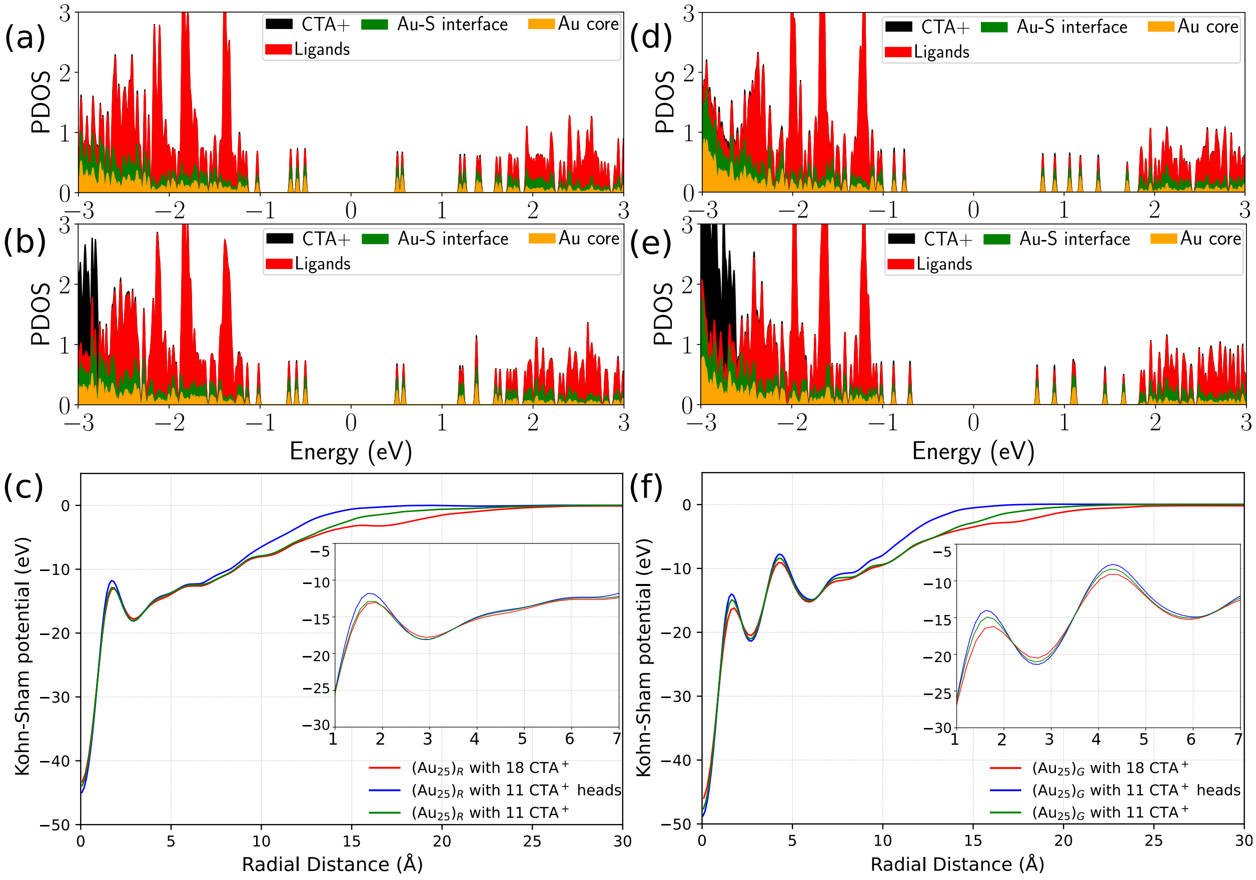

Figure S2: Partial densities of states (PDOS) for $(Au_{25})_R$ (a,b)and $(Au_{25})_G$ (d,e) under isomers with 11 TMA$^+$(a,d) and with 11 CTA$^+$(b,e). Calculated Kohn–Sham radial potentials, $V_{KS}(r)$, for (c) $(Au_{25})_R$ and (f) $(Au_{25})_G$

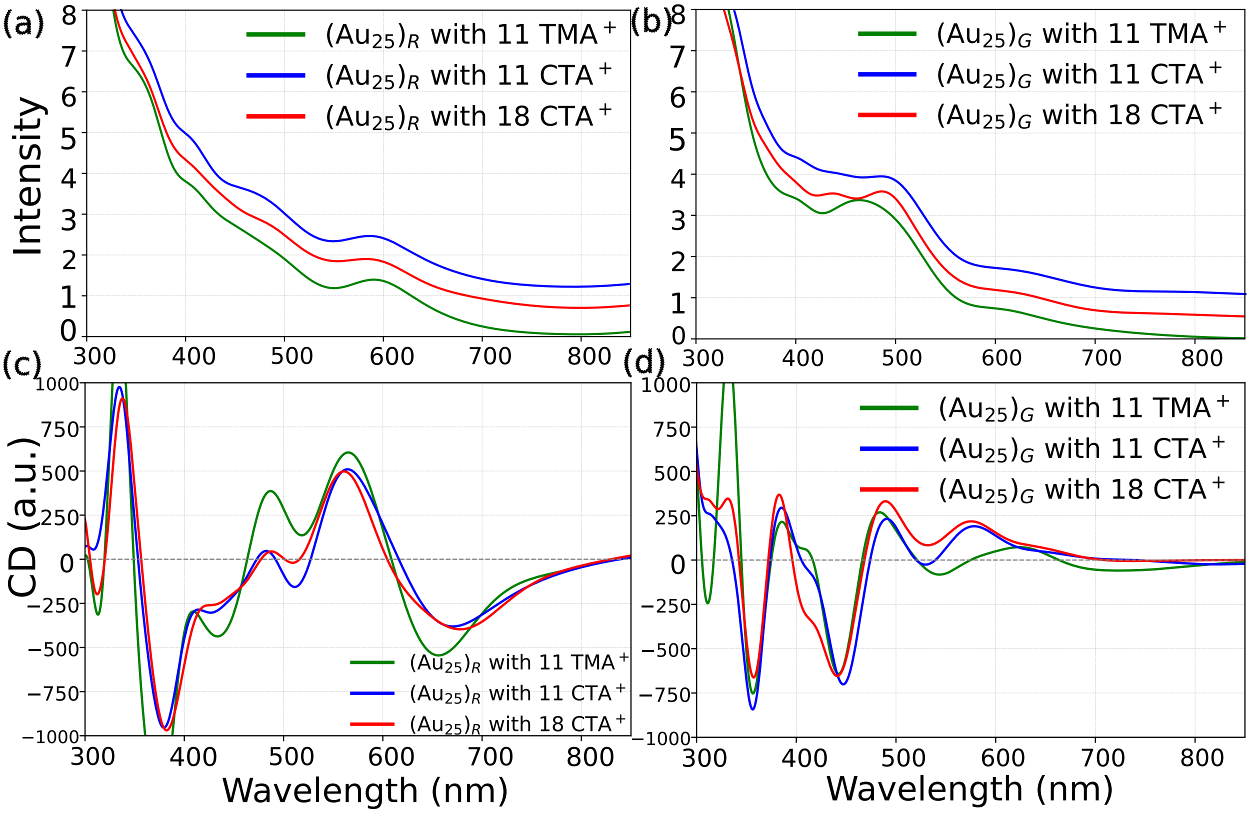

Figure S3: Calculated optical absorption spectra and circular dichroism (CD) spectra for (a) $(Au_{25})_R$ and (b) $(Au_{25})_G$ isomers under isomers with 11 TMA^+, with 11 CTA^+, and with 18 CTA^+.
